# Heterologous prion-forming proteins interact to cross-seed aggregation in *Saccharomyces cerevisiae*

**DOI:** 10.1038/s41598-017-05829-5

**Published:** 2017-07-19

**Authors:** Kathryn M. Keefer, Kevin C. Stein, Heather L. True

**Affiliations:** 1 Department of Cell Biology and Physiology, Washington University School of Medicine, St. Louis, Missouri 63110 United States of America; 2 Department of Biology, Stanford University, Stanford, California United States of America

## Abstract

The early stages of protein misfolding remain incompletely understood, as most mammalian proteinopathies are only detected after irreversible protein aggregates have formed. Cross-seeding, where one aggregated protein templates the misfolding of a heterologous protein, is one mechanism proposed to stimulate protein aggregation and facilitate disease pathogenesis. Here, we demonstrate the existence of cross-seeding as a crucial step in the formation of the yeast prion [*PSI* ^+^], formed by the translation termination factor Sup35. We provide evidence for the genetic and physical interaction of the prion protein Rnq1 with Sup35 as a predominant mechanism leading to self-propagating Sup35 aggregation. We identify interacting sites within Rnq1 and Sup35 and determine the effects of breaking and restoring a crucial interaction. Altogether, our results demonstrate that single-residue disruption can drastically reduce the effects of cross-seeding, a finding that has important implications for human protein misfolding disorders.

## Introduction

The aggregation of heterologous proteins is implicated in many human neurodegenerative diseases, including Parkinson’s disease, Alzheimer’s disease, and amyotrophic lateral sclerosis (ALS). These fatal conditions are characterized by progressive neuronal damage and functional decline resulting from the co-aggregation of diverse, disease-related proteins^1^. Though the catastrophic effects of protein aggregation have been well-described, less is known about the factors that contribute to the initial formation of heterologous protein inclusions. Recent findings suggest that a cross-seeding mechanism, whereby one protein aggregate stimulates the misfolding of another, may induce the aggregation of heterologous proteins and have a profound impact on disease onset and progression^2–5^. However, while implicated, direct mechanistic evidence of cross-seeding has yet to be shown.

Yeast prions, like pathogenic mammalian amyloids, are beta sheet-rich aggregate structures that can be transmitted from cell to cell. They are a robust model for studying the aggregation of human proteins, as yeast prions can also form varying conformational “strains” that impart differing cellular phenotypes in a manner analogous to the distinct structures and pathologies formed by amyloidogenic proteins in humans^6–9^. Indeed, the study of yeast prions has resulted in significant contributions to elucidating fundamental mechanisms of protein misfolding^10–12^. The yeast prion protein Sup35 has provided a unique model for probing the nature of heterologous cross-seeding. Sup35 is a translation termination factor of *Saccharomyces cerevisiae* that aggregates to form the prion known as [*PSI* ^+^]^13^. Interestingly, *de novo* [*PSI* ^+^] formation is largely dependent upon the presence in the cell of another yeast prion, [*RNQ* ^+^], that has no known function outside of its influence on [*PSI* ^+^]^10, 14^. Yet, like the co-aggregation of mammalian proteins, the nature of the relationship between Sup35 and Rnq1 remains unclear.

**Figure 1:**
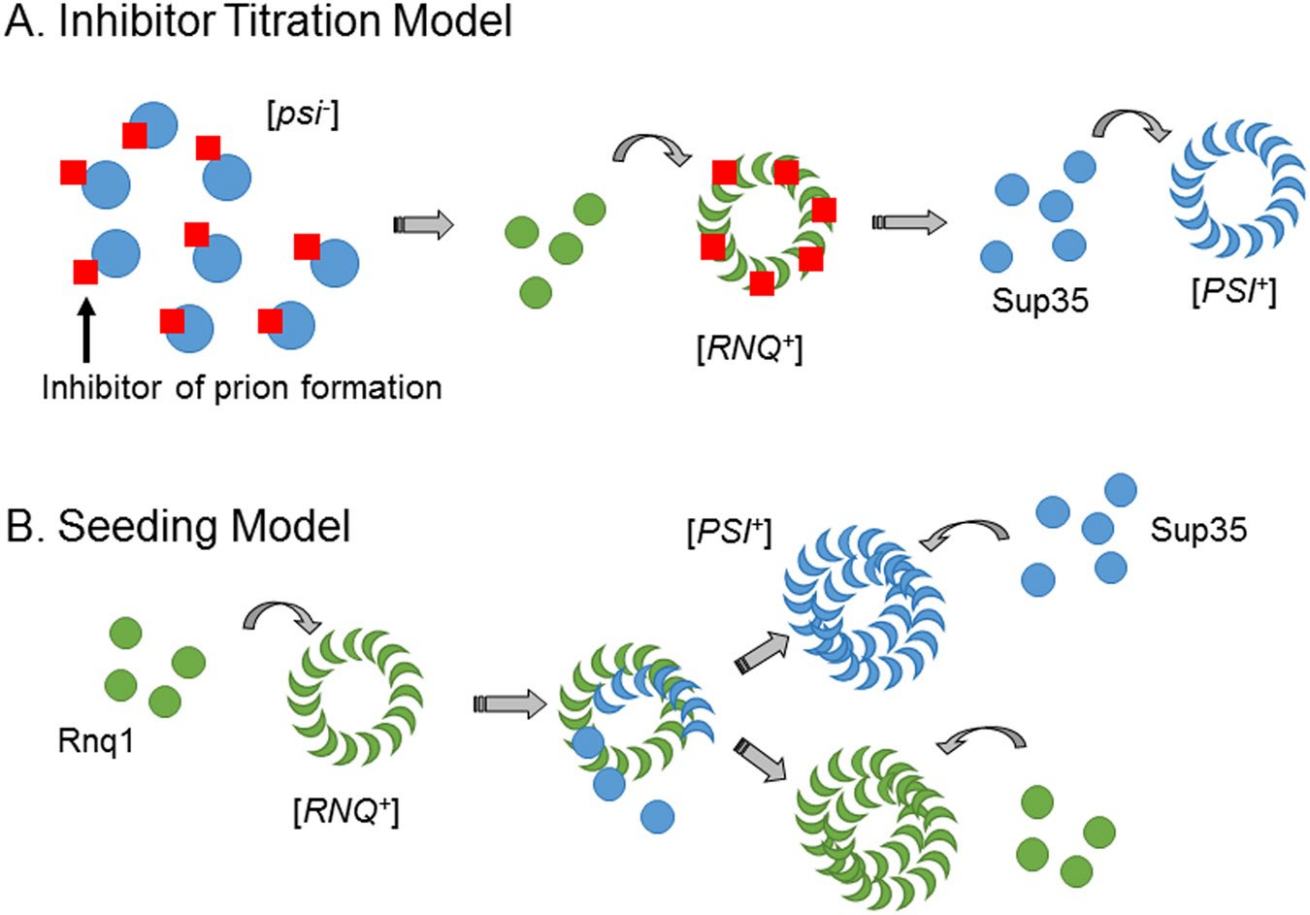
Models for the [*RNQ* ^+^]-dependent formation of the [*PSI* ^+^] prion. (**A**) The inhibitor titration model suggests that an inhibitor molecule (red squares) binds to Sup35 in its soluble state (blue circles). The presence of [*RNQ* ^+^] (green ring) sequesters the inhibitor away from Sup35, thereby allowing the protein to aggregate and form [*PSI* ^+^]. (**B**) The seeding model suggests that there is a physical interaction between Sup35 and aggregated Rnq1 during the formation of [*PSI* ^+^]. After the interaction, the [*RNQ* ^+^] and [*PSI* ^+^] prions are propagated independently.

There are two predominant models for the dependence of Sup35 on [*RNQ* ^+^] to form [*PSI* ^+^] (Fig. 1)^10, 15–17^. The inhibitor titration model posits the existence of an inhibitor molecule that binds to monomeric Sup35 and prevents its aggregation in [*rnq* ^−^] cells (Fig. 1A). Upon formation of [*RNQ* ^+^], this inhibitor is sequestered by the Rnq1 aggregates and titrated away from Sup35, leaving Sup35 free to aggregate to form [*PSI* ^+^]. The putative inhibitor has been suggested to be a molecular chaperone or an element of the ubiquitin-proteasome machinery^18^. Previous work, including an extensive screen, attempted to identify an inhibitor^16^, but no candidate molecules were revealed.

In contrast to the titration model, the cross-seeding model suggests that Sup35 and Rnq1 physically interact during [*PSI* ^+^] formation (Fig. 1B). In this manner, the misfolded and aggregated Rnq1 in the [*RNQ* ^+^] state induces Sup35 to misfold via bending the monomer out of its native conformation or templating a spontaneously misfolded monomer into a structure that is conducive for prion propagation. There is some evidence for the seeding model. Fluorescence microscopy demonstrated that newly-forming Sup35-YFP aggregates co-localized with existing Rnq1-CFP aggregates^19^. Promoting the interaction between Rnq1 and Sup35 via a Sup35-Rnq1 fusion protein also enhanced [*PSI* ^+^] formation, whereas fusion of Sup35 to other aggregation-prone molecules did not^20^. In addition, preformed aggregates of the Rnq1 prion-forming domain (PFD) have been shown to slightly increase the rate of Sup35 polymerization^19, 21–23^, and Rnq1 weakly co-immunoprecipitates with Sup35 *in vivo* ^22^.

To gain greater insight into the aggregation of heterologous proteins, we set out to investigate the nature of the Sup35-Rnq1 interaction. Using a variety of techniques *in vivo* and *in vitro*, we have demonstrated a binding interaction that supports the veracity of the seeding model. We probed the functional consequences of disrupting this binding, and for the first time in any system, identified sites of interaction that are responsible for cross-seeding and facilitating the spread of aggregation between two heterologous amyloidogenic proteins.

## Methods

### Yeast strains, plasmids, cultures, and transformations

Yeast strains were cultured using standard techniques^24^. Yeast transformation with plasmid DNA was performed by the PEG/LiOAC method^24^. All yeast strains used were 74-D694. Complete lists of strains and plasmids are available in Tables S1 and S2, respectively.

### Protein purification

Sup35 constructs and Rnq1 were tagged with a C-terminal polyhistidine tail and purified on nickel affinity resin. Briefly, BL21 (DE3) bacteria were transformed with the plasmid of interest and grown at 37 °C in LB+ Amp media. At OD_600_ = 0.6, protein expression was induced with 1 mM IPTG and cultures were grown at 25 °C for 6 hours. Cells were pelleted, washed, and frozen at −80 °C until use. Cells were thawed at 4 °C for 2hr, shaken in lysis buffer (Sup35: 20 mM Tris, 0.5 M NaCl, 5 mM imidazole, 2 M urea, 0.1% IGEPAL; Rnq1: 20 mM Tris, 8 M Urea, pH 8) at 4 °C for 30 minutes, and then sonicated 5 × 10 seconds at high power. Lysates were cleared by centrifugation at 20,000 × g for 30 minutes, and the supernatant was incubated with a nickel resin for 30 minutes at 4 °C. Resin columns and bound proteins were washed with washing buffer (Sup35: 20 mM Tris-HCl, 0.5 M NaCl, 20 mM imidazole, 2 M urea; Rnq1: 20 mM Tris, 8 M Urea, pH 6.3–5.9) and eluted with wash buffer + 400 mM imidazole for Sup35 or wash buffer at pH 4.5 for Rnq1. Elution fractions were pooled, concentrated, and methanol-precipitated prior to use. Rnq1 purification was performed as described previously^25^.

### Rnq1-Trap

His-tagged Sup35 was expressed in BL21 (DE3) cells and partially purified as described above, but retained bound to the nickel resin. Yeast cultures were grown to mid-log phase and mechanically lysed with acid-washed glass beads (Sigma) in Sup35 binding buffer + protease inhibitors (20 mM Tris, 0.5 M NaCl, 5 mM imidazole, 2 M urea, 0.1% IGEPAL, 50 mM NEM, 3 mM PMSF, 1 tablet Roche Protease Inhibitor Cocktail #4693159001). Cleared lysates were incubated for 30 minutes with the Sup35-bound nickel resin prior to washing with 150 mL washing buffer (20 mM Tris-HCl, 0.5 M NaCl, 20 mM imidazole, 2 M urea) and elution with 100 mL elution buffer (20 mM Tris-HCl, 0.5 M NaCl, 400 mM imidazole, 2 M urea). Fraction collection was followed by standard SDS-PAGE (10% polyacrylamide) and western blotting.

### Thioflavin T kinetics

Purified Sup35NM and Rnq1 were resuspended to 300 µM in 7 M guanidine hydrochloride. Rnq1 fibers were formed at 4 °C, 25 °C, and 37 °C and then pooled. Fiber formation and kinetic assays were performed under agitation with acid-washed glass beads (Sigma) as previously described^26^, with minor alterations to buffers (Sup35: 5 mM KPO_4_, 150 mM NaCl, pH 7.4; Rnq1: 50 mM KPO_4_, 2 M Urea, 150 mM NaCl, pH 6).

### Boiled gels

Yeast strains were cultured overnight in selective media. Cells were lysed in buffer (25 mM Tris-HCl pH 7.5, 50 mM KCl, 10 mM MgCl_2_, 1 mM EDTA, 10% glycerol, protease inhibitors) by mechanical disruption with acid-washed glass beads (Sigma). Lysates were normalized by total protein and mixed with SDS-PAGE sample buffer (200 mM Tris-HCl pH 6.8, 4% SDS, 0.4% bromophenol blue, 40% glycerol). Unboiled samples were loaded on a 12% polyacrylamide gel and run under 140 V of current until the dye front migrated halfway through the resolving gel. The current was stopped and the gel and glass plates were sealed in plastic sheets prior to boiling upright for 15 minutes in a 95 °C water bath. The gels were removed from the plastic and reinserted into the PAGE apparatus, where voltage was re-applied (140 V) until the dye front migrated to the bottom of the gel. This procedure was followed by standard western blotting with Sup35 and Rnq1 antibodies (Supplementary Table S3).

### Prion manipulation and protein biochemistry

[*PSI* ^+^] induction and SDD-AGE experiments were performed as previously described^8, 27^. [*PSI* ^+^] induction frequency was calculated by counting the number of white or white-sectored colonies on each plate and dividing by the total number of colonies on the plate. Cytoduction was performed as previously described^28, 29^.

### Screen for Sup35 mutations

The screen for suppressor mutations was performed using SP5, a chimeric Sup35 protein that contains 5 repeat domains from the prion protein PrP in place of the endogenous repeat domains of Sup35^30, 31^. SP5 was utilized in the screen because it is a robust inducer of [*PSI* ^+^]^30^. To introduce mutations, six error-prone PCR reactions were performed in the presence of 25 µM MnCl_2_ using Taq polymerase and 35 cycles to amplify the NM domains of *SP5*. PCR reactions were combined to create three separate libraries, followed by SpeI/BclI digestion and ligation into p415*TEF-SP5*. 74-D694 *sup35Δ rnq1Δ* yeast cells propagating m.d. high [*RNQ* ^+^] and harboring episomal copies of *SUP35* and *rnq1-Q298R* were transformed with each library, with selection on SD-His-Leu. Transformation plates were replica plated onto SD-His-Leu + 5-FOA to select for cells having replacement of the *URA3*-marked *SUP35* plasmid. These colonies were replica plated onto YPD and YPD^+^3 mM GdnHCl to identify white [*PSI* ^+^] colonies that were also curable. More than ~3,000 colonies were assessed, of which 193 were pink (putative [*PSI* ^+^]) and 38 of those were curable (suggestiong the prion). After plasmid recovery and sequencing, we identified 12 mutant *SP5* alleles.

### Crosslinking

PCR was performed on pAED4-*SCNM-his7* to introduce the G7C mutation, using forward primer 5′GCGCATATGATGTCGGATTCAAACCAATGTAACAATCAGCAAAAC (mutated bases underlined) and reverse primer 5′CATCGATGAATTCTTAATGGTGG to amplify the His-tagged NM domain and introduce the mutation (underlined bases). The PCR product and pAED4 vector were digested with NdeI and EcoRI, ligated, and transformed into BL21 (DE3) cells for protein expression and purification as described above. Following methanol precipitation, Sup35NM-G7C and Rnq1 were dissolved to 0.1 mM in conjugation buffer (PBS, 2 mM EDTA, pH 7.4). The heterobifunctional crosslinker SMPB (Thermo #22416) was attached to Rnq1 by incubating 1.3 mg protein with a final concentration of 1 mM SMPB for 30 minutes at room temperature. Disulfide bonds in Sup35NM-G7C were reduced with TCEP (Sigma). Excess SMPB and TCEP were removed by passing proteins through Zeba desalting columns (Thermo #89890). Equimolar amounts of Sup35-G7C and Rnq1-SMPB were combined and incubated at room temperature for 30 minutes, followed by SDD-AGE and western blotting.

### Data availability

All data generated or analyzed during this study are included in this article or are available from the corresponding author.

## Results

### Rnq1 aggregates seed the polymerization of Sup35

We set out to specifically test the cross-seeding model, which posits a physical interaction between Rnq1 and Sup35. We began examining the interaction between Rnq1 and Sup35 by assessing the kinetics of *in vitro* aggregation using Thioflavin T (ThT)^32^. ThT binds specifically to amyloid fibers, and an increase in the fluorescent emission signal indicates formation of amyloid. When incubated with agitation at room temperature, the prion forming domain of Sup35, denoted Sup35NM, will form amyloid after a reproducible lag phase^25, 31^. This lag phase can be reduced or eliminated if Sup35NM aggregation is “seeded” by preformed Sup35NM fibers that provide a template for amyloid formation^25^. Previous work has demonstrated that the Rnq1 prion-forming domain (PFD) can increase Sup35NM polymerization, albeit inefficiently^19, 21, 22^. As “self-seeding” (i.e. propagating [*PSI* ^+^]) *in vivo* is more efficient than cross-seeding (i.e. inducing [*PSI* ^+^] in [*RNQ* ^+^] cells), this is somewhat expected. Indeed, self-seeding of Sup35NM *in vitro* with pre-formed Sup35NM fibers is much more efficient than heterologous seeding^21^. However, we now know that regions outside of the Rnq1 PFD are important for maintenance of the [*RNQ* ^+^] prion, and that the distinct conformations of Rnq1 aggregates propagated by different [*RNQ* ^+^] prion strains, or variants, dramatically affect the formation of [*PSI* ^+^] *in vivo* ^7, 21–23, 28, 33–36^. Thus, we utilized full-length Rnq1 and aimed to create a mixed population of seed conformers that could emulate the aggregation states that may be present *in vivo*.

**Figure 2:**
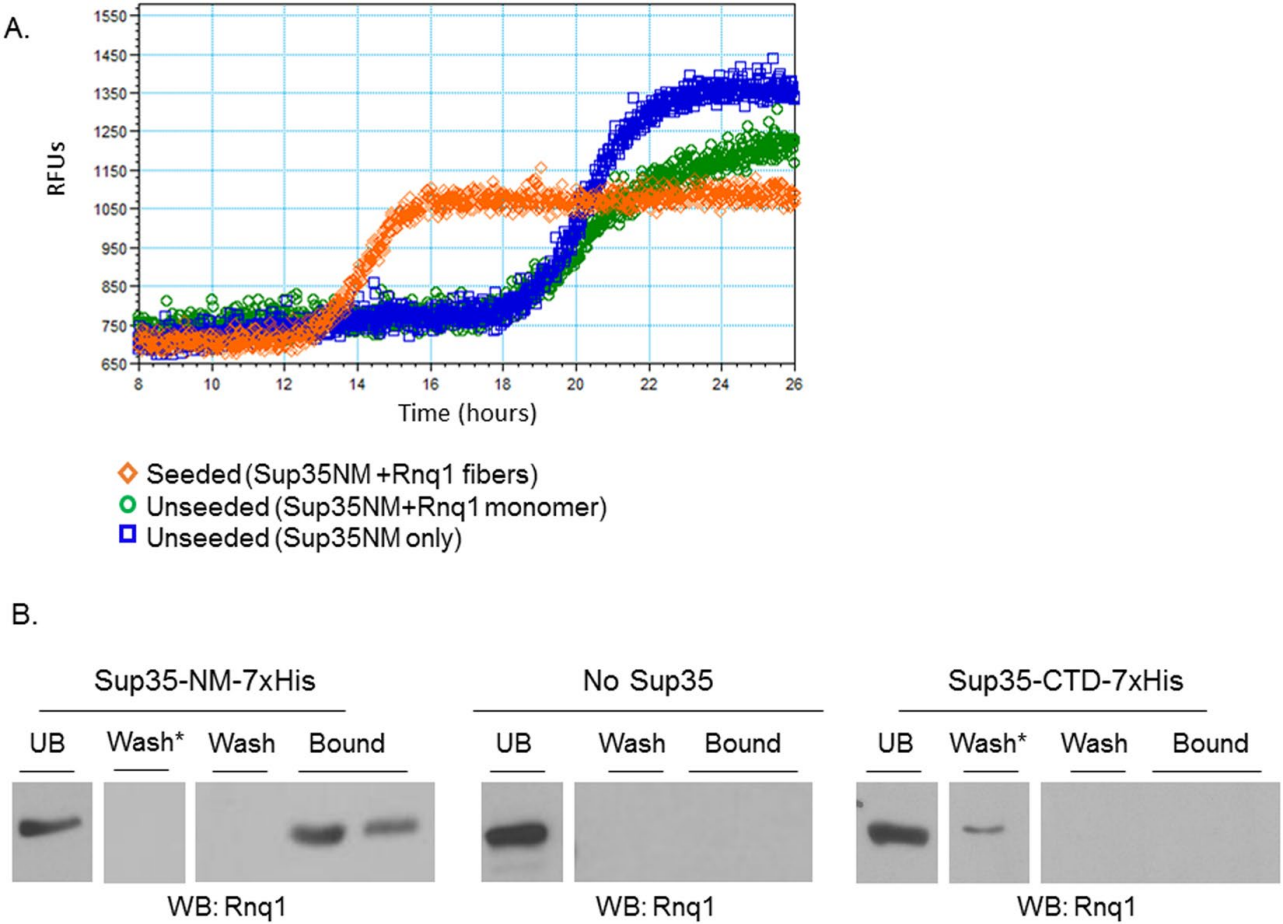
Rnq1 and Sup35 physically interact. (**A**) ThT kinetic experiments monitor the polymerization of amyloid via enhanced fluorescent emission. Unseeded Sup35 (blue line) polymerizes and aggregates after approximately 17 hours, as does Sup35 incubated with Rnq1 monomer alone (green line). The addition of Rnq1 fibers (orange line) reduces the lag time for Sup35 polymerization to approximately 12.5 hours. Curves represent data from three experiments. (**B**) Sup35 was immobilized on resin and utilized as bait for a [*RNQ* ^+^] trap assay. Cell lysates were incubated with the resin, washed with buffer, and eluted. The presence of trapped, untagged Rnq1 was detected via western blot with anti-Rnq1 antibody. Lanes shown are the unbound (UB) fraction, the final wash fraction and the first two elution fractions. “Wash*” indicates an intermediate wash fraction. Blots are representative images from three independent experiments.

We purified recombinant Sup35NM and full-length Rnq1, and formed Rnq1 fibers *in vitro* to serve as “seeds” for the polymerization reaction. As we previously demonstrated that temperature can affect the conformation of Rnq1 aggregates, such as changing the amount of fiber branching and bundling^26^, we formed Rnq1 fibers at a range of temperatures and combined them to create a broad pool of potential seed structures. Thermal stability experiments confirmed the existence of Rnq1 aggregates (not shown). We then performed ThT aggregation assays with monomeric Sup35NM in both unseeded and seeded conditions (Fig. 2A). We found that the addition of monomeric Rnq1 did not have an effect on the lag phase of Sup35 polymerization relative to the unseeded reaction. However, the presence of pooled Rnq1 seeds at 4% (v/v) concentration reduced the lag phase of Sup35NM aggregation as predicted (Fig. 2A). Thus, the initiation of Sup35 aggregation *in vitro* is enhanced by the presence of Rnq1 amyloid. As this experiment used pure, recombinant protein and Rnq1 seeds were sufficient to enhance Sup35 polymerization, these results support the cross-seeding model (Fig. 1).

### Sup35 stably binds Rnq1 *ex vivo*

We hypothesized that the conformation of Rnq1 in [*RNQ* ^+^] cell lysates may differ from that readily obtained in the *in vitro* amyloid formation reaction. Therefore, we sought to test the binding of Sup35 to a biologically relevant conformation of Rnq1 isolated from [*RNQ* ^+^] yeast cell lysates. To do so, we bound recombinant, His-tagged Sup35 containing either the NM prion-forming domain or the non-prion C-terminal domain (CTD) to nickel affinity resin. We then prepared cell lysates from [*RNQ* ^+^][*psi* ^−^] yeast and incubated the lysate with the nickel resin to allow for binding between Rnq1 and immobilized Sup35. We hypothesized that the bound Sup35NM would “trap” untagged Rnq1 that would ordinarily flow through the column. We washed the column with increasing amounts of imidazole to displace the bound proteins, and collected unbound, wash, and elution (bound) fractions (Fig. 2B). We found that the Sup35 CTD-7xHis experienced weak or non-specific binding to Rnq1, as the detectable Rnq1 appeared predominantly in the unbound and intermediate wash fractions. However, there was a strong interaction between Rnq1 and Sup35 when the Sup35NM-7xHis protein was used as bait, as a large proportion of Rnq1 appeared in the bound fractions. Rnq1 was still present in the unbound fraction, yet not all Rnq1 is expected to be aggregated or Sup35-binding-competent in [*RNQ* ^+^] cells. Importantly, when no Sup35NM-7xHis was present on the beads, all Rnq1 protein was present in the unbound fraction, indicating the lack of non-specific binding of Rnq1 to the column. Thus, the strong binding between *ex vivo* Rnq1 to the Sup35 prion-forming domain appears to be specific and stable.

### The rnq1-Q298R mutation causes a [PSI^+^] induction defect

**Figure 3:**
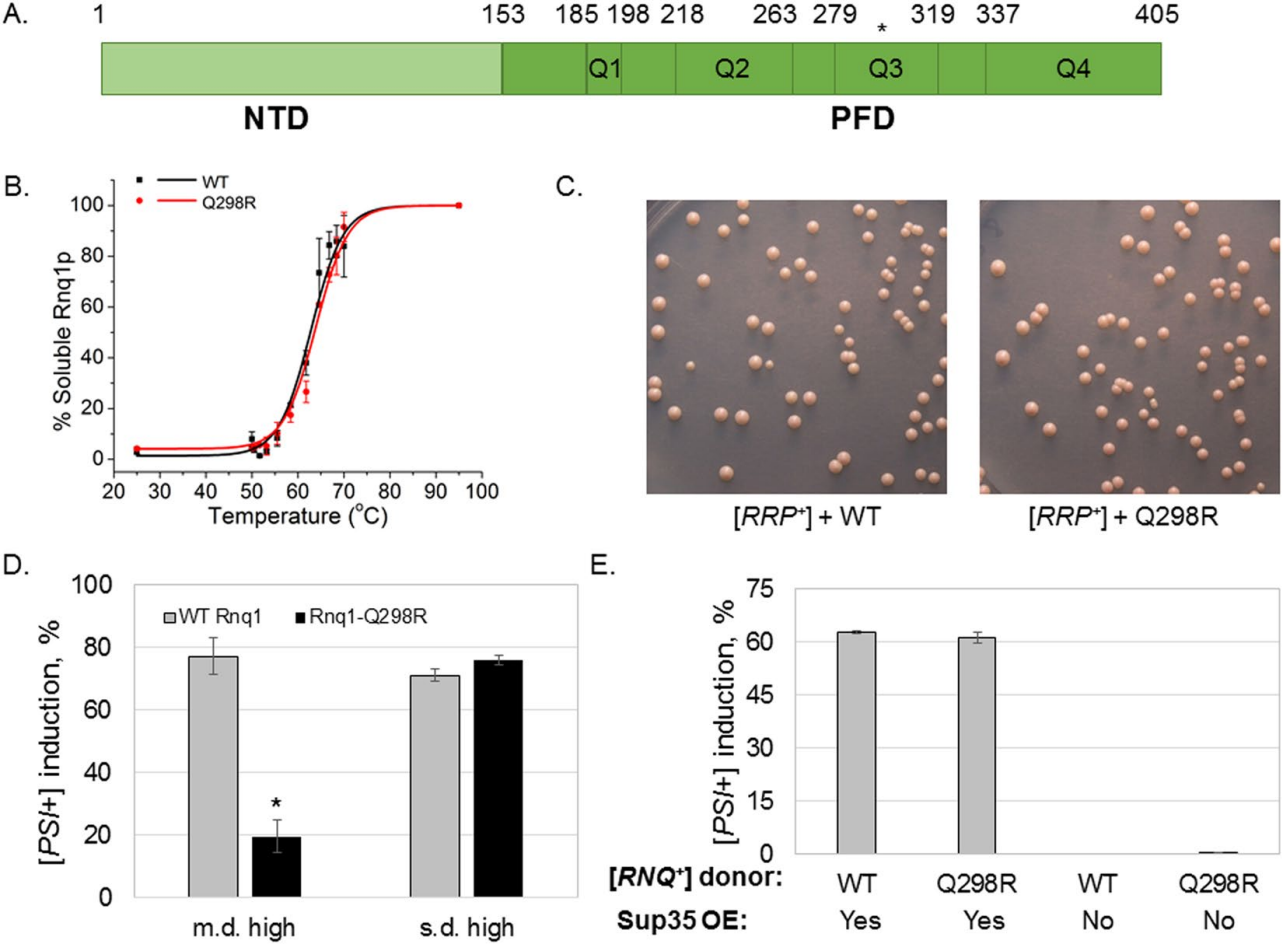
Rnq1-Q298R causes a [*PSI* ^+^] induction defect. (**A**) The Rnq1 protein contains an N-terminal domain (NTD) and a glutamine/asparagine-rich prion-forming domain (PFD). The PFD contains four glutamine-dense regions, Q1-4^36^. The Q298R mutation occurs in region Q3 of the PFD. (**B**) Rnq1 aggregates were treated at a gradient of temperatures in SDS to determine the melting point of the Rnq1-Q298R aggregates versus the WT. There were no significant differences in the thermostable properties of either protein aggregate. Western blot signal from multiple experiments was quantified using ImageJ, normalized by the 95C band, and plotted using Origin 9.0. Error bars represent standard error of the mean (s.e.m). (**C**) There are no detectable differences in mitotic stability of Rnq1-Q298R aggregates as compared to WT Rnq1 aggregates. [*RNQ* ^+^] strains containing the [*R* *NQ* ^+^] Reporter Protein (RRP), a phenotypic readout for the [*RNQ* ^+^] prion^37^, were transformed with plasmids harboring either WT *RNQ1* or *rnq1-Q298R*. We assessed the mitotic stability, or spontaneous prion loss, of resulting strains and found that [*RNQ* ^+^] formed from WT or Rnq1-Q298R was similarly maintained. (**D**) The *rnq1-Q298R* mutant shows a strong defect in [*PSI* ^+^] induction relative to WT cells of the m.d. high variant of [*RNQ* ^+^]. [*PSI* ^+^] was induced by overexpression of Sup35 in [*psi* ^−^][*RNQ* ^+^] cells of either a *RNQ1* or *rnq1-Q298R* genetic background. Colonies were assessed by color, with white or pink colonies or sectored colonies scored as [*PSI* ^+^]. Error bars represent mean ± s.e.m. The “_*_” symbol represents a significant difference between [*PSI* ^+^] induction in *RNQ1* versus *rnq1-Q298R* backgrounds, p < 0.001. (**E**) Results from [*PSI* ^+^] induction following cytoplasmic transfer of either WT Rnq1 or Rnq1-Q298R protein aggregates into a [*rnq* ^−^] *RNQ1* strain. The propagated [*RNQ* ^+^] would be templated from the WT or mutant aggregate, but be comprised of only WT protein. Error bars represent mean ± s.e.m.

Upon demonstrating the physical interaction between Sup35 and Rnq1, we sought to identify the specific residues in each protein that were important to binding. To do so, we utilized a mutation in Rnq1, Q298R, that had been previously identified by our lab and demonstrated to impart a [*PSI* ^+^] induction defect (Fig. 3A)^37^. This work was performed with the “multi-dot high” variant of [*RNQ* ^+^] that is a strong inducer of [*PSI* ^+^]. The mutant was previously shown to be capable of propagating [*RNQ* ^+^]^37^, and our additional tests of thermal stability (Fig. 3B, Supplementary Figure S1) and mitotic stability (Fig. 3C) showed that the Rnq1-Q298R mutant maintains Rnq1 aggregates that are biochemically indistinguishable from WT Rnq1.

Though they propagate [*RNQ* ^+^] efficiently, cells harboring *rnq1-Q298R* were shown to have a [*PSI* ^+^] induction defect; that is, they become [*PSI* ^+^] at a much lower frequency than *RNQ1* cells^37^. To verify this defect, we utilized a colorimetric assay to visualize the frequency of [*PSI* ^+^] induction *in vivo*. The colorimetric readout relies upon a well-described phenotypic [*PSI* ^+^] reporter, using an *ade1-14* genetic background, which impacts adenine production in a manner that depends on nonsense suppression. Since Sup35 is a translation termination factor, its aggregation to form [*PSI* ^+^] increases nonsense suppression. The *ade1-14* allele contains a premature stop codon in the *ADE1* gene^38^. Yeast containing *ade1-14* cannot make adenine and colonies grown on media containing adenine appear red due to the buildup of a pigmented metabolic byproduct. The presence of [*PSI* ^+^] allows cells to read through the *ade1-14* premature stop codon and synthesize adenine. As such, [*PSI* ^+^] cells can grow on synthetic media lacking adenine (SD-Ade) and appear white or pink on rich media plates (YPD), whereas [*psi* ^−^] colonies are red.

Utilizing this colorimetric test to monitor [*PSI* ^+^] induction, we overexpressed Sup35 in [*RNQ* ^+^][*psi* ^−^] yeast expressing either Rnq1 or Rnq1-Q298R. To build upon our previous work, we examined two [*RNQ* ^+^] prion variants – multi-dot high (m.d. high) and single-dot high (s.d. high) – in order to assess the specificity of the mutant, which was previously characterized in only m.d. high. Cells were spread on rich media plates to allow for development of red or white colonies. The presence of productive Rnq1-Sup35 interactions would induce the formation of white [*PSI* ^+^] colonies. In agreement with our previous results, strains with WT Rnq1 aggregates induced [*PSI* ^+^] at a high level in both variants, whereas yeast with Rnq1-Q298R aggregates exhibited a significant decrease in [*PSI* ^+^] formation in the m.d. high strain (Fig. 3D). In combination with our observation that there is no detectable effect of Rnq1-Q298R on aggregate structure, this suggests that Q298 is an important site of interaction between Rnq1 and Sup35, and that mutating the glutamine residue leads to decreased or unproductive interactions with Sup35. Interestingly, *rnq1-Q298R* had no effect upon [*PSI* ^+^] induction in conjunction with the s.d. high [*RNQ* ^+^] variant (Fig. 3D), suggesting that the residues involved in cross-seeding differ between different prion strains. As our lab has demonstrated that distinct regions of Rnq1 are important for the propagation of different [*RNQ* ^+^] variants^35^, it is not surprising that there are also differences in the regions important for Sup35 interaction.

We then sought additional confirmation that the [*PSI* ^+^] induction defect of *rnq1-Q298R* was indeed related to the genetic mutation and not a heritable change in the [*RNQ* ^+^] prion conformation. If the [*RNQ* ^+^] aggregate conformation was heritably altered as a result of the Rnq1-Q298R mutation, then we would expect to see a [*PSI* ^+^] induction defect in WT cells that had been infected with Rnq1-Q298R aggregates. To test this, we utilized cytoduction to transfer aggregates from a [*RNQ* ^+^]^Q298R^ donor cell to a [*rnq* ^−^]^WT^ recipient cell expressing *RNQ1*. Thus, the recipient yeast would become [*RNQ* ^+^] and propagate the prion conformation from the donor strain, but with WT Rnq1 protein. Following cytoduction from either [*RNQ* ^+^]^WT^ or [*RNQ* ^+^]^Q298R^ donors to a [*rnq* ^−^]^WT^ recipient, we monitored [*PSI* ^+^] induction and found that the *RNQ1* recipient strains receiving WT Rnq1 and Rnq1-Q298R aggregates induced [*PSI* ^+^] to equivalent levels (Fig. 3E). Thus, the [*PSI* ^+^] induction defect in *rnq1-Q298R* is a consequence of the genetic mutation as opposed to a templating defect that is transmitted to WT Rnq1.

### Suppressor mutations rescue the [PSI^+^]-induction defect

**Figure 4:**
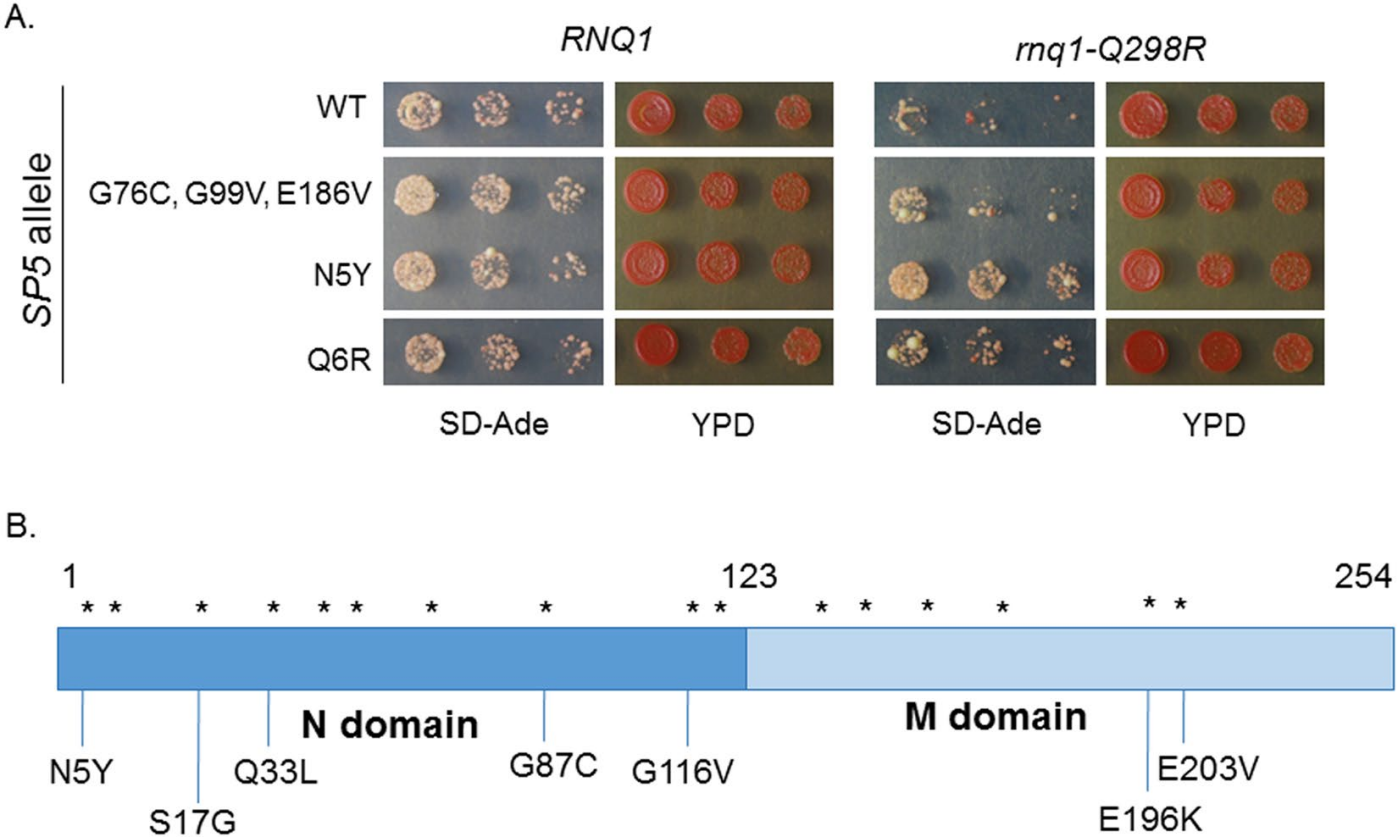
Sup35 mutations can restore interaction with Rnq1-Q298R. (**A**) Candidates from a suppressor screen to identify Sup35 mutants that can rescue the [*PSI* ^+^] induction defect associated with *rnq1-Q298R*. These mutants enhanced [*PSI* ^+^] formation in the *rnq1-Q298R* genetic background relative to WT *SP5*. Horizontal white bars separate non-adjacent spottings from the same plate. Complete spottings of screen candidates appear in Supplementary Figure S2. (**B**) The indicated mutations were cloned into *SUP35* for further testing.

After confirming the effect of *rnq1-Q298R* on [*PSI* ^+^] induction, we sought to identify compensatory mutations in Sup35 that might represent putative binding sites between Rnq1 and Sup35. We performed a second site suppressor screen to identify mutations within the Sup35 prion forming domain that could restore the interaction with Rnq1-Q298R and rescue the [*PSI* ^+^] induction defect. Briefly, we transformed strains containing *rnq1-Q298R* with a pool of mutagenized *SP5*, a chimeric *SUP35* construct with high [*PSI* ^+^] induction rates that allowed us to resolve fine differences between the rescuing abilities of putative suppressor mutations^30^. We then assessed the ability of yeast cells to form [*PSI* ^+^] (see *Methods* for screen details). Our secondary tests verified that [*PSI* ^+^] was formed by showing that the nonsense suppression phenotypes were reversible on plates containing 3 mM GdnHCl, which cures yeast prions^39^ (not shown). To assess [*PSI* ^+^] formation, we spotted the screen candidates onto SD-Ade to test their growth (Fig. 4A, Supplementary Figure S2A). The most promising mutants were those that grew strongly on SD-Ade relative to WT *SP5* in the *rnq1-Q298R* genetic background. In all, we isolated 12 *sup35* mutants containing a total of 16 nonsynonymous mutations within the “NM” prion-forming domain (Fig. 4B).

### Sup35-N5Y robustly rescues the [PSI^+^]-induction defect

**Figure 5:**
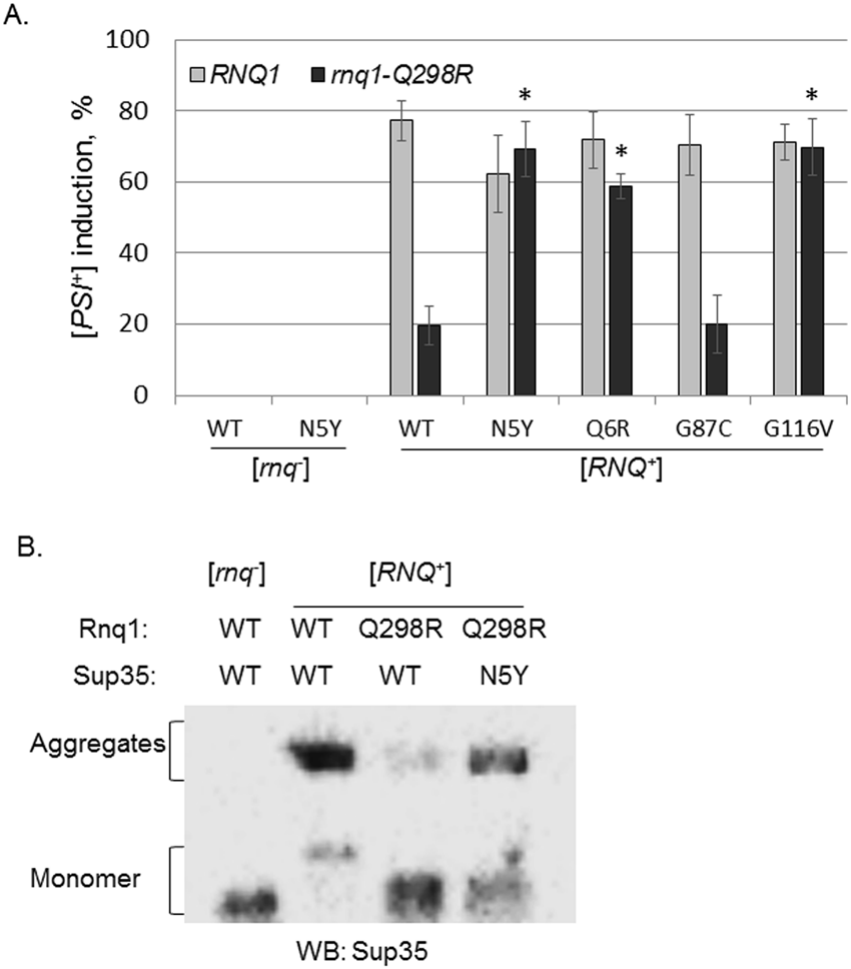
Sup35-N5Y strongly rescues the [*PSI* ^+^] induction defect. (**A**) [*PSI* ^+^] induction experiments demonstrate that three Sup35 mutants restore [*PSI* ^+^] formation to nearly WT levels in *rnq1-Q298R* cells. These mutations do not increase [*PSI* ^+^] formation in a *RNQ1* genetic background. Horizontal axis labels denote *SUP35* genetic status. The [*rnq* ^−^] control cells express *RNQ1* and *SUP35*. More than 13,000 colonies were assessed over five biological replicates. Error bars represent mean ± s.e.m. The “*” symbols represent a significant difference between [*PSI* ^+^] induction with WT Sup35 in *rnq1-Q298R* versus induction with the indicated *sup35* mutants, p < 0.005. (**B**) Boiled gel assays allow separate migration of monomeric and aggregated materials, confirming that Sup35 aggregation is reduced in the *SUP35 rnq1-Q298R* background relative to *SUP35 RNQ1* cells. Sup35 aggregation is restored in *sup35-N5Y rnq1-Q298R* cells. We attribute the slightly aberrant monomer bands as an artifact of the experimental procedure, as samples were unboiled (and likely non-denatured) as per the protocol, and Sup35 may shed unevenly from aggregates in addition to the soluble pool. Western blot is a representative image from three independent experiments.

After cloning the 16 individual candidate mutations from *SP5* into *sup35*, we then tested their ability to suppress the [*PSI* ^+^] induction defect of *rnq1-Q298R*. We again utilized [*PSI* ^+^] induction experiments to evaluate the *sup35* screen candidates (Fig. 5A and not shown). We overexpressed either WT or mutant Sup35 in [*RNQ* ^+^] cells expressing either Rnq1 or Rnq1-Q298R from integrated alleles covering endogenous *RNQ1*. Both strains were *sup35Δ* and expressed the C-terminal domain of Sup35 to allow for shuffling of the mutant constructs. Importantly, [*PSI* ^+^] formation was never observed in [*rnq* ^−^] cells, and a high degree of induction occurred in WT [*RNQ* ^+^] cells. Moreover, the [*PSI* ^+^] induction defect was evident in [*RNQ* ^+^] *rnq1-Q298R SUP35* cells. However, three Sup35 mutants rescued the defect and induced [*PSI* ^+^] to near-WT levels: N5Y, Q6R, and G116V (Fig. 5A, Supplementary Figure S2B). The G87C mutation, which was isolated in combination with G116V (Fig. 4A), did not restore [*PSI* ^+^] induction (Fig. 5A). Interestingly, despite M domain mutations also being identified and tested, the three rescuing mutations all fall within the N-terminal region of Sup35, which is the more aggregation-prone domain thought to be the most crucial for prion formation^17, 40^.

There were two adjacent single mutants isolated during our screen, *sup35-N5Y* and *sup35-Q6R*. Since *sp5-N5Y* had the strongest growth on SD-Ade (Fig. 4A and Supplementary Figure S2B), we decided to focus on the potential interaction between Sup35-N5 and Rnq1-Q298. First, we examined Sup35 aggregation biochemically via “boiled gel” assays^41^. In these experiments, unboiled cell lysates are loaded on SDS-PAGE gels. Native (non-denatured) aggregated material is too large to enter the resolving gel and becomes trapped in the wells and the stacking gel. Upon boiling the gel midway through electrophoresis, aggregated material breaks down and enters the resolving gel. Following western blots, two bands are visible per lane: a lower band of soluble material, and a higher band of insoluble material that was delayed in entering the gel (see *Methods*). We performed boiled gel experiments with cell lysates generated from overnight cultures of [*RNQ* ^+^][*psi* ^−^] cells containing either WT *RNQ1* or *rnq1-Q298R* and overexpressing WT or *sup35-N5Y* (Fig. 5B, Supplementary Figure S3). We also examined a [*rnq* ^−^] control with WT *RNQ1* and *SUP35*. As expected, the [*rnq* ^−^] control showed no Sup35 aggregation, since [*PSI* ^+^] rarely forms in the absence of [*RNQ* ^+^]. In WT cells, nearly all of the Sup35 was visualized in the aggregated band, indicating prolific [*PSI* ^+^] formation. Conversely, little aggregated Sup35 was detected in cells expressing *rnq1-Q298R* in conjunction with *SUP35*, mirroring the [*PSI* ^+^] induction defect. Finally, in *rnq1-Q298R* cells harboring the *sup35-N5Y* mutation, a dramatic increase in Sup35 aggregation was observed. The lack of complete restoration of Sup35 aggregation may be due to incomplete rescue by *sup35-N5Y*, or the shorter yeast culture period for this protocol (16 hours) versus [*PSI* ^+^] induction experiments (4 days), potentially indicating that Rnq1-Q298R propagates a sufficient yet non-optimal template for seeding [*PSI* ^+^] formation relative to WT Rnq1. Collectively, our results demonstrate that the Sup35 N5Y mutation rescues Sup35 aggregation and [*PSI* ^+^] formation when [*RNQ* ^+^] is propagated by Rnq1-Q298R.

### The Sup35 N-terminus binds to Rnq1 *in vitro*

**Figure 6:**
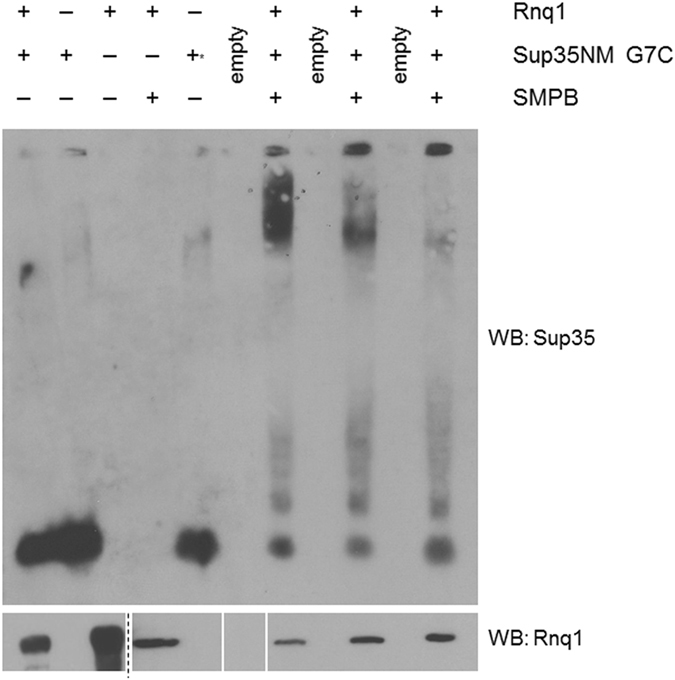
The N terminal region of Sup35 crosslinks to Rnq1. Site-directed crosslinking of Sup35-G7C to Rnq1 created high-molecular weight aggregates as visualized by SDD-AGE. Rnq1 alone, Sup35-G7C alone, and both proteins together without SMPB did not create large aggregates. The “^+^*” notation in the fifth lane indicates Sup35 monomer following treatment with TCEP. Western blot is a representative image from three independent experiments. In the lower blot, Rnq1 loading was confirmed by SDS-PAGE. Vertical white bars separate non-adjacent lanes of the same blot. The dashed line separates adjacent lanes of the same blot under differing film exposures for image clarity (full blots in Supplementary Figure S4). There is excess Rnq1 in lanes 1 and 3, samples prepared without crosslinker, to adjust for protein that is lost during sample desalting following attachment of the crosslinker (see *Methods*). Three trials of crosslinking were included in each experiment and the crosslinking experiment was repeated with different batches of purified proteins three times.

After demonstrating that Sup35 and Rnq1 physically interact and that Rnq1-Q298 and Sup35-N5 may be an important site of contact, we sought to verify the interaction by crosslinking *in vitro*. We decided to use a sulfhydryl-reactive crosslinker to take advantage of the lack of cysteine residues in Sup35. We chose to introduce a cysteine mutation close to the putative binding site so as not to disturb the residue(s) identified as being important in the interaction (N5 and the adjacent screen candidate Q6). We mutated the Sup35 G7 residue to cysteine to direct crosslinking to the N-terminal region of the protein. The G7C mutation of Sup35 has previously been shown to form and propagate the [*PSI* ^+^] state in a manner identical to WT Sup35^42^. After treating recombinant Rnq1 with a heterobifunctional crosslinker, SMPB, the two proteins were combined and analyzed via semi-denaturing detergent agarose gel electrophoresis (SDD-AGE), a protocol that allows for the visualization of large SDS-resistant aggregated species (Fig. 6). We utilized SDD-AGE so that we could visualize the full spectrum of outcomes of the Sup35-Rnq1 interaction; that is, the induction of additional aggregation. We also confirmed the presence of crosslinked heterodimers via SDS-PAGE (Supplementary Figure S4).

In all crosslinked reactions, a very high molecular-weight product was visible in the wells of the gel. This product reacted with both Sup35 and Rnq1 antibodies (Fig. 6 and Supplementary Figure S4), and was not present in control lanes containing Rnq1 alone, Sup35NM-G7C alone, or the two proteins combined without SMPB. Based on the spacer arm length of SMPB (11.5 Å), this indicates that Rnq1 and Sup35 physically bind within the first 15 amino acids of Sup35, and agrees with the genetic interaction found in our second site suppressor screen.

## Discussion

The early events of protein misfolding and aggregation in human diseases have been difficult to elucidate using mammalian systems. A longstanding question has been whether interactions between heterologous proteins might contribute to the onset and progression of disease. Taking advantage of the tractable yeast prion model, we have utilized biochemical and genetic techniques to demonstrate the physical interaction of heterologous prion-forming proteins *in vitro* and *in vivo*. Utilizing a combination of genetic techniques and crosslinking, we identified specific amino acid residues that are important for the binding of Sup35 and Rnq1; namely, the N5 residue of Sup35 and the Q298 residue of Rnq1. This indicates that the prion-forming domains of these proteins interact to transmit the prion state between distinct proteins. Many disease-associated proteins also have aggregation-prone regions that could facilitate promiscuous interactions with other proteins to facilitate their own aggregation^43, 44^.

Importantly, although our work strongly supports the “seeding” model of Rnq1-Sup35 interaction, it does not disprove the inhibitor titration model (Fig. 1). It may be that a combination of both models will best explain the *de novo* formation of [*PSI* ^+^] *in vivo*. For instance, an inhibitor molecule might be titrated away from Sup35 to allow for the physical interaction between Rnq1 aggregates and Sup35 that our data show is necessary for [*PSI* ^+^] formation. The inhibitor itself, having affinity for both Sup35 and Rnq1, may even facilitate the Sup35-Rnq1 binding while it is being titrated by Rnq1. Molecular chaperones have been suggested as potential inhibitor molecules^18^. In support of this concept, previous research by our lab and others has demonstrated that several classes of chaperones can have opposing effects upon prion formation and propagation, sometimes in a conformation-dependent manner^45–47^. It is possible that the Rnq1 monomer itself may have an inhibitory effect upon Sup35 aggregation. Future research may implicate one or more of these proteins as an inhibitor of [*PSI* ^+^] formation. However, our results suggest that cross-seeding is sufficient for Sup35 polymerization *in vitro* and is clearly necessary for [*PSI* ^+^] formation *in vivo*.

In addition to showing the importance of a physical interaction for [*PSI* ^+^] formation, our data demonstrate that the extreme N-terminus of Sup35 plays a critical role in facilitating this interaction. As the Sup35 N-terminal domain lacks a defined structure, it may be that this region of the protein is most freely available to interactions with other proteins. The charged M domain might then act as a stabilizer for whichever conformation is adopted by the disordered N domain. However, beyond the N-terminus, we uncovered other mutations that could partially rescue the [*PSI* ^+^] induction defect associated with Rnq1-Q298R (Fig. 4). This suggests that there are multiple residues where Sup35 and Rnq1 may interact *en route* to Sup35 aggregation. Though each of the three investigated Sup35 mutations was alone sufficient to restore interaction with Rnq1-Q298R, there may be multiple points of contact necessary before Sup35 assumes its prion conformation. By directing an interaction between Sup35 and Rnq1 at residues of interest (via a dimerizing tag or crosslinking agent), it may be possible to drive very high levels of interaction and subsequent aggregation *in vivo*. The total number of necessary interactions and their order of occurrence (if any) remain avenues of further investigation.

It is also likely that heterologous interactions of amyloidogenic proteins depends on the structure of the aggregate, or prion strain. Different prion strains adopt different tertiary protein conformations^48^. Various data suggest that these conformational differences result from beta sheets being formed either from distinct amino acid residues or from alternative packing of the same residues, thereby presenting different points of contact^48, 49^. Our previous work indicated that the regions that form the Rnq1 aggregate β-sheets of the different [*RNQ* ^+^] variants may be distinct^35^. As we showed that these differences affected aggregate-chaperone interactions^47^, so might the intermolecular contacts with Sup35 be different. Indeed, our work with the multi-dot high variant of [*RNQ* ^+^] presented here identified Q298 as an important binding site with Sup35, but Q298R had no apparent effect on Sup35-Rnq1 interaction in the “single-dot high” prion strain (Fig. 3D). This suggests that the Rnq1 aggregates propagated in single-dot high [*RNQ* ^+^] cells might have a different set of binding residues with Sup35. While prion strains likely have distinct contact points, there may also be a cohort of interacting sites that remain consistent between prion strains. Such putative differences in contact of Rnq1 and Sup35 between the different [*RNQ* ^+^] variants might help explain their drastically different abilities to induce the formation of [*PSI* ^+^].

Furthermore, the [*RNQ* ^+^] strain present in a cell influences the distribution of [*PSI* ^+^] strains that form^22, 27^. This phenomenon may also be explained by the existence of different interaction sites between the two prion-forming proteins. In our experiments, we observed our Sup35 mutants forming various strains of [*PSI* ^+^] under both WT and *rnq1-Q298R* genetic backgrounds (not shown). Thus, this suggests that the Sup35-N5 and Rnq1-Q298 residues comprise a key binding site that supports normal [*PSI* ^+^] formation with multi-dot high [*RNQ* ^+^]. Other sites of interaction may influence strain specificity. This would provide mechanistic detail for how distinct amyloid conformers can influence the types of aggregate structures formed by heterologous proteins, which might impact the progression of human diseases.

Indeed, many human pathologies involve the aggregation of misfolded, beta sheet-rich proteins^50^. Although these incurable diseases have profound effects upon patients and a multi-billion dollar impact on the healthcare system^51^, little is known about the factors that cause the *de novo* protein misfolding. It is possible that a seeding or templating mechanism contributes to the misfolding and subsequent aggregation of human proteins. In support of this concept, the Parkinson’s disease-related protein α-synuclein has been shown to cross-seed the fibrillization of tau, which is implicated in Alzheimer’s disease pathology^4^. Further, polymerization of amyloid-beta (Aβ) was recently shown to be promoted by the presence of misfolded islet amyloid polypeptide (IAPP), which aggregates in the islet cells of patients with type 2 diabetes^5^. This is intriguing in light of epidemiological studies that have identified type 2 diabetes as a risk factor for Alzheimer’s disease^52^. While our work demonstrates that multiple binding sites may be involved in cross-seeding (Fig. 4), it also suggests that blocking one crucial site might be sufficient to alter the outcomes of the interaction. Further studies in yeast might provide a foundation for developing more focused therapeutic approaches to target these crucial interaction sites.

## Electronic supplementary material

Supplementary Tables and Figures
