# Supplementary material for: Heterologous prion-forming proteins interact to cross-seed aggregation in Saccharomyces cerevisiae
Source: Sci Rep. 2017 Jul 19;7:5853. doi: 10.1038/s41598-017-05829-5 (PMC5517628; doi:10.1038/s41598-017-05829-5)
Supplement: Supplementary file 1 — Supplementary Tables and Figures [file 41598_2017_5829_MOESM1_ESM.pdf]

Heterologous prion-forming proteins interact to cross-seed aggregation in *Saccharomyces cerevisiae*

Kathryn M. Keefer,[1] Kevin C. Stein,[2] and Heather L. True[1]*

Supplementary Information

Table S1. Strains used in this study.

Strain	Description	Reference
2261	74D-694; [*psi*-], high [*RNQ*+], *MATα, ade1-14 his3Δ-200 trp1-289 ura3-52 leu2-3,112*, *sup35Δ::HygBMX4*, *RNQ1*	30
2160	74D-694; [psi-], high [RNQ+], *MATα, ade1-14 his3Δ-200 trp1-289 ura3-52 leu2-3,112*, *sup35Δ::HygBMX4*, *RNQ1-Q298R*	This study
1890	74D-694; [*psi*-], [*rnq*-], *MATA, ade1-14 his3Δ-200 trp1-289 ura3-52 leu2-3,112*, *sup35Δ::HygBMX4*, *RNQ1*	30
1951	74D-694; [*psi*-], [*rnq*-], *MATA, ade1-14 his3Δ-200 trp1-289 ura3-52 leu2-3,112*, *sup35Δ::HygBMX4*, *RNQ1-Q298R*	This study
2040	74D-694; [*psi*-], [*rnq*-], *MATα, ade1-14 his3Δ-200 trp1-289 ura3-52 leu2-3,112*, *sup35Δ::HygBMX4*, *kar1-Δ15*, ρ0	This study
2047	74D-694; [*psi*-], high [*RRP*+], *MATA, ade1-14 his3Δ-200 trp1-289 ura3-52 leu2-3,112*, *sup35Δ::RMC, rnq1Δ::KANMX4*	30
1282	74D-694; [*psi*-], high [*RNQ*+], *MATA, ade1-14 his3Δ-200 trp1-289 ura3-52 leu2-3,112*, *sup35Δ::KANMX4, rnq1Δ::KANMX4*	This study

Table S2. Plasmids used in this study.

Plasmid	Description	Use	Reference
5049	pYK810	Cover *SUP35* deletion with *SUP35*	50
5281	p416*TEF-SP5*	Template for screen	This study
5787	p415*TEF-SP5*	Backbone for screen candidate expression	This study
6244	pEMBL-*SUP35*	[*PSI*+] induction – WT	30
6257	pRS315-*sup35-N5Y*	Cover *SUP35* deletion with *sup35-N5Y*	This study
6342	pEMBL-*sup35-N5Y*	[*PSI*+] induction – N5Y	This study
6473	pRS315-*sup35-Q6R*	Cover *SUP35* deletion with *sup35-Q6R*	This study
6483	pEMBL- *sup35-Q6R*	[*PSI*+] induction – Q6R	This study
6474	pRS315-*sup35-G116V*	Cover *SUP35* deletion with *sup35-G116V*	This study
6484	pEMBL- *sup35-G116V*	[*PSI*+] induction – G116V	This study
5074	pPROEX-Htb-*RNQ1*	WT Rnq1 purification	This study
SL7111	pAED4-*SCNM-his7*	WT Sup35 bait for Rnq1-trap on resin	14
6771	pAED4-*SCNM-G7C-his7*	Purification for crosslinking reagent	This study
6772	pAED4-*SCTD-his7*	Control bait for Rnq1-trap on resin	This study
5719	pRS313-*rnq1-Q298R*	Mitotic stability testing of Rnq1-Q298R	This study
6312	pRS313-*RNQ1*	Mitotic stability testing of WT Rnq1	30

Table S3. Antibodies used in this study.

Antigen	Production notes	Type	Dilution	Source
Sup35	Peptide, a.a.s 137-151	Rabbit polyclonal	1:1,500	Lindquist lab
Rnq1	Full-length Rnq1	Rabbit polyclonal	1:1,000	True lab
Rnq1	Full-length Rnq1	Rat polyclonal	1:2,000	True lab
Rabbit	Rabbit IgG	Goat polyclonal	1:10,000	Sigma A0545
Rat	Rat IgG	Rabbit polyclonal	1:10,000	Sigma A5795

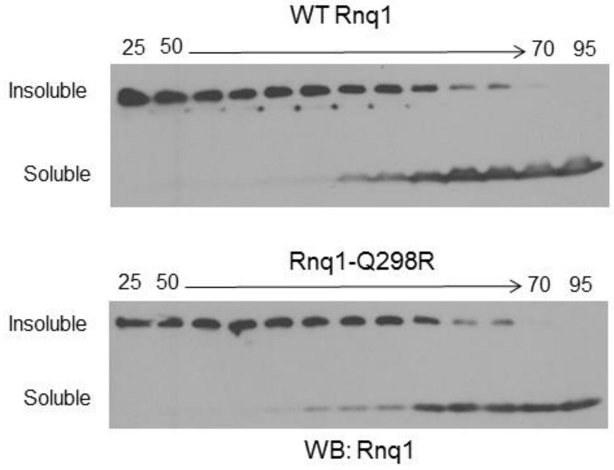

Figure S1. Rnq1-Q298R aggregates have similar properties to WT Rnq1 aggregates
Representative western blots from thermal stability experiments quantified in Figure 3B. Cell lysates were subjected to a temperature gradient before SDS-PAGE and western blotting. Insoluble material, such as prion aggregates, requires treatment at high temperatures in order to enter the resolving gel. There were no observable differences between WT and Rnq1-Q298R aggregates.

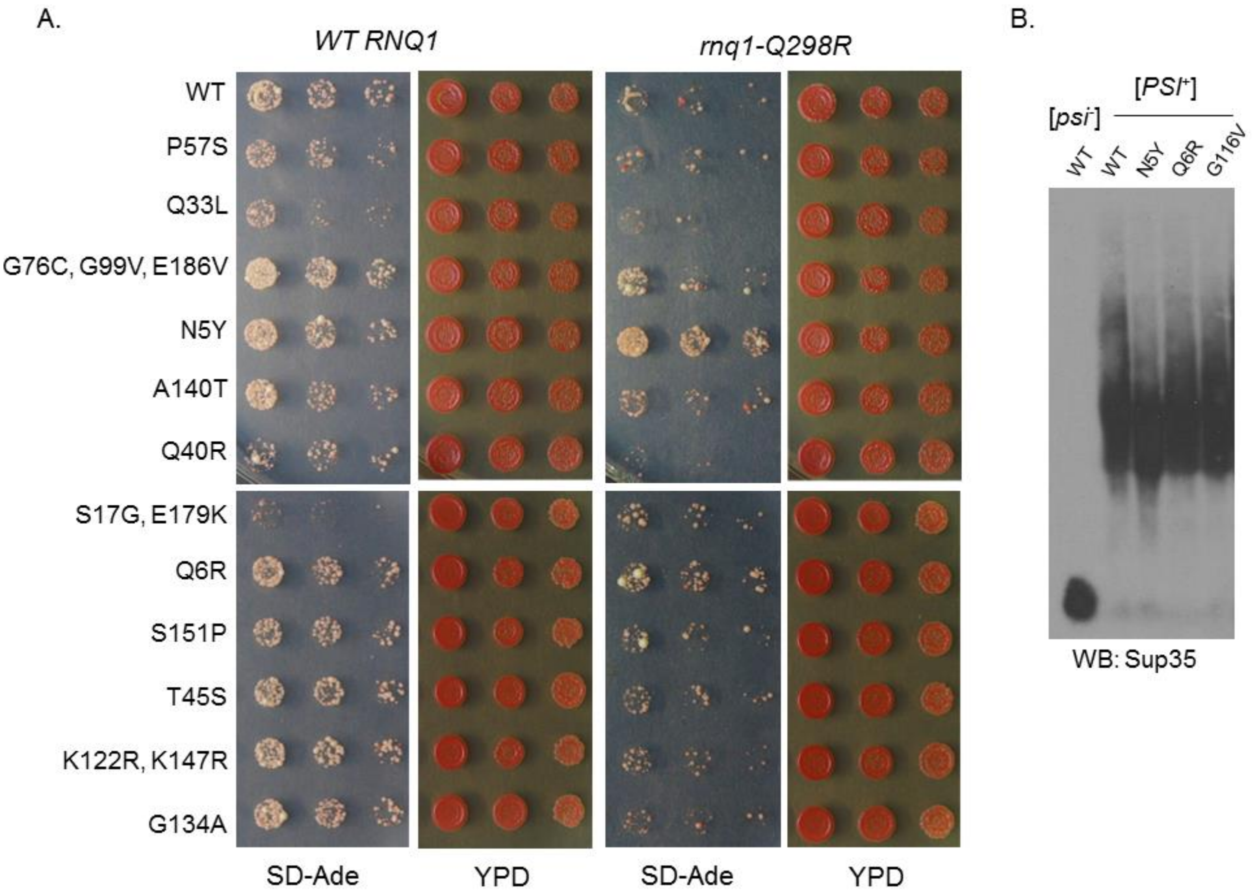

Figure S2. Mutants isolated from *SP5* suppressor screen

(A) Full spottings of screen candidates from Figure 4A. (B) SDD-AGE analysis demonstrates that the rescuing mutants Sup35-N5Y, Sup35-Q6R, and Sup35-G116V can all propagate [*PSI*$^+$].

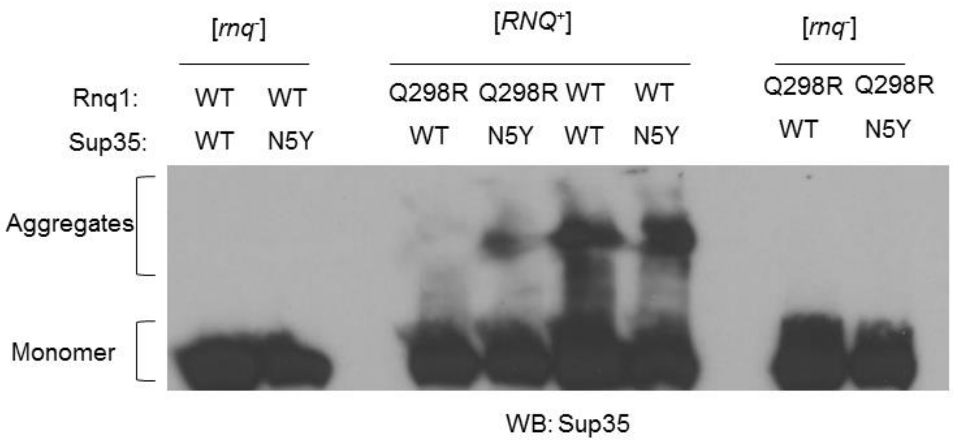

Figure S3. Boiled gel assay with additional [*rnq*⁻] controls

As in Figure 5B, the indicated yeast strains we subjected to a boiled gel protocol to assess the aggregated vs monomeric Sup35. No aggregated Sup35 was detectable in any of the [*rnq*⁻] strains, regardless of genetic background.

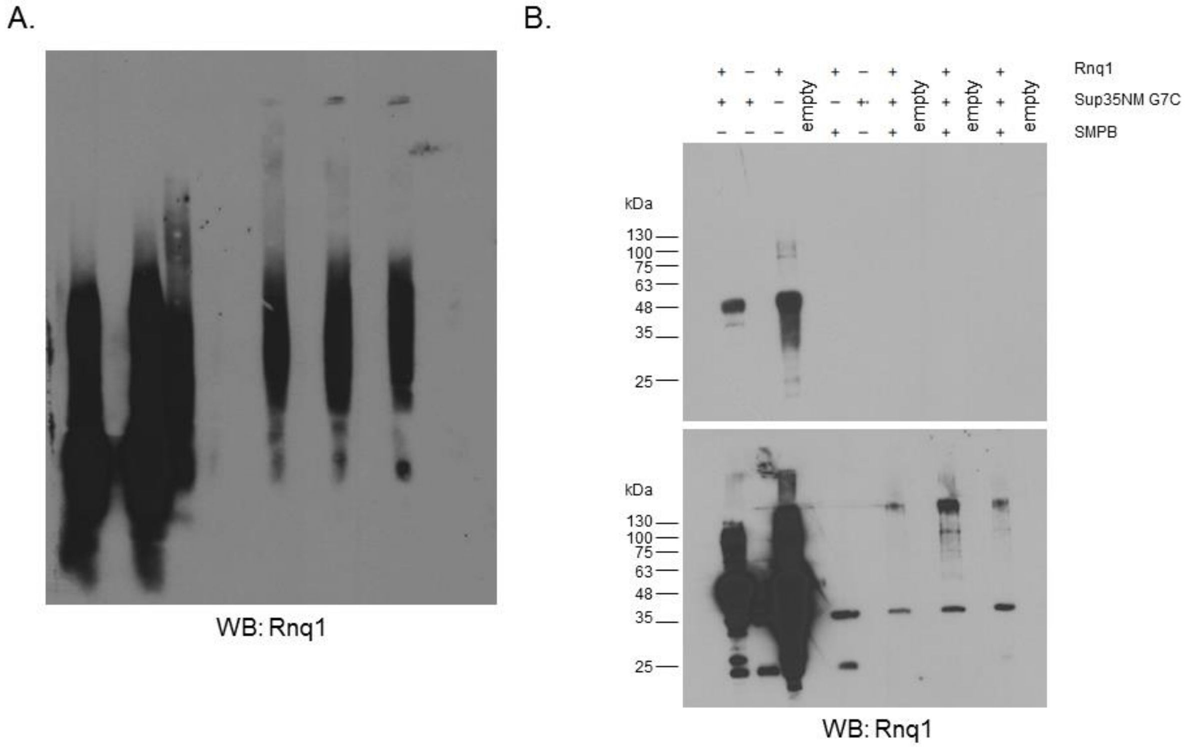

Figure S4. Full crosslinking blots

(A) SDD-AGE from Figure 6, probed with rat anti-Rnq1 primary and rabbit anti-rat secondary antibodies. (B) Complete SDS-PAGE blot from Figure 6. Two exposures are shown to allow for clear viewing of the recombinant Rnq1 in lanes 1 and 3.
